# Burnout, Sleep, and Sleepiness during Day and Night Shifts in Transition from 8- to 12-Hour Shift Rosters among Airline Ground Crew Managers

**DOI:** 10.3390/clockssleep1020020

**Published:** 2019-04-24

**Authors:** Tamar Shochat, Satanay Hadish-Shogan, Michal Banin Yosipof, Ayelet Recanati, Orna Tzischinsky

**Affiliations:** 1 The Cheryl Spencer Department of Nursing, Faculty of Social Welfare and Health Sciences, University of Haifa, Haifa 3498838, Israel; 2 Haemek Hospital, Afula 18317, Israel; 3 El Al Israeli Airlines LTD., Ben Gurion Airport 7015001, Israel; 4 Behavioral Sciences, Max Stern Yezreel Academic College, Emek Yezreel 19300, Israel

**Keywords:** sleep quality, sleepiness, burnout, shiftwork, shift length, napping, caffeine

## Abstract

Organizational changes in shift scheduling provide rare opportunities for field studies aimed at investigating the effects of such changes on health and wellbeing. We studied the effects of a transition from 8-hour (8-h) to 12-hour (12-h) shift rosters in 39 airline ground crew managers on burnout, sleep quality, and sleepiness. Assessments were collected during the 8-h and were repeated three months after the transition to 12-h shift rosters. These assessments included the Shirom-Melamed Burnout Measure (SMBM), the Pittsburgh Sleep Quality Index (PSQI), actigraphy, the Karolinska Sleepiness Scale (KSS) completed hourly during one day and two night shifts, and caffeine intake. Findings demonstrated lower burnout, improved sleep quality, improved quality of naps, and increased afternoon sleepiness during the 12-h day shift. Napping was reported during 12-h night shifts by 36% of the sample. In nappers, increased night shift sleepiness was associated with increased caffeine intake on 8- and 12-h shifts. In non-nappers, increased night shift sleepiness was associated with decreased caffeine intake on the 8-h shift only. Change in shift length affects other structural and behavioral parameters in the workplace, making it challenging to isolate distinct characteristics of the two rosters and their relative effects on study outcomes. Individual differences in adaptation to shiftwork may also play a role.

## 1. Introduction

For the past several decades, ergonomics researchers have attempted to optimize shiftwork schedules in order to improve health, safety, welfare, and functional outcomes for shift workers. Factors studied in recent years that have been shown to affect sleep, sleepiness, burnout, and other health and functional outcomes include type of shift (e.g., fixed afternoon/evening, rotating [1,2], short rest periods between shifts [3,4], unpredictable work hours [4], split shifts [4,5], and shift length [6,7]. In the present investigation, we were offered an opportunity to prospectively examine functional outcomes in the context of a planned pilot transition from 8-hour (8-h) to 12-hour (12-h) shift rosters in shift-working ground crew airline managers. 

Studies focusing on the length of the shift within the 24-hour day have typically contrasted 8-h and 12-h shifts [6,7,8,9,10,11]. However, few field studies have prospectively assessed the health and functionality of shift workers transitioning from 8- to 12-h shift rosters; these have revealed different and often inconsistent findings [10,12,13,14,15,16]. In a series of studies using the National Institute for Occupational Safety and Health (NIOSH) fatigue and performance test battery in control room operators [13,14] and in employees of a natural gas utility [15], findings consistently showed decline in performance tasks that were attributed to the extra work hours and shorter sleep duration following the transition from 8-h to 12-h shifts. Assessment of performance over consecutive workdays revealed that some performance tasks (e.g., grammatical reasoning response time and accuracy) improved across days in the 12-h but not in the 8-h shift rosters, suggesting that the shorter workweek may offset some of the negative effects of the longer work shift [13]. Investigators concluded that the extra work hours per shift and the associated sleep debt and increase in fatigue explain the decline in performance during the 12-h shifts, and that despite this decline, the opportunity to condense the work week into fewer shifts is popular among workers who are willing to endure the increased fatigue and compromised performance [15]. 

Other studies demonstrated some functional improvements when moving from 8- to 12-h shift rosters in measurements including blood pressure, sleep duration, sleep quality, and subjective level of alertness [12], psychological health and fatigue, [16], mood, sleep and general health measures, as well as higher satisfaction regarding domestic and social domains [10]. However, higher error rates were recorded on performance tests towards the end of the 12-h but not the 8-h shifts [10]. Beyond the apparent diversity in workers’ professions and in the measured outcomes, we cautiously conclude that workers report improved outcomes regarding their health and wellbeing despite indications of compromised performance. It is important to note that typically, in these studies, only a subset of workers participate in all measurements for both the 8-h and 12-h shifts (e.g., [10,14,15]). Therefore, data analysis was based on independent between-group designs. 

Few studies have assessed the effects of shift length on burnout, fatigue, job stress, and job satisfaction, and these were based on cross-sectional studies of hospital nurses [6,17,18]. In a European study, a shift length of 12 hours or more was associated with a 26% increase in reported emotional exhaustion, a 21% increase in experiences of depersonalization, and a 39% decrease in sense of personal accomplishment. Nurses reported less satisfaction with work and schedule flexibility and were more likely to report an intention to leave their job as compared to a shift length of 8-h or less [6]. Similar findings were reported in a study of U.S. nurses, showing an increase in burnout and job dissatisfaction with increasing shift lengths [17]. 

Conversely, one study found lower emotional exhaustion and increased job and schedule satisfaction in nurses working 12-h compared to 8-h shifts [19]. The urban setting or other demographic characteristics of the sample in this study may explain these discrepant findings. In addition, typical characteristics of hospital nurses, including female gender and associated family responsibilities, low job control, and high psychological demands are likely to contribute to job strain, burnout, and job satisfaction [20]. For this reason, the subjects may not be comparable to managerial workers in other less stressful settings. 

It is evident from the literature that most of the studies that examined the effects of shift length (8-h versus 12-h) compared independent groups of workers or prospectively followed small sample sizes. Very few field studies have been performed on the same individuals transferring from 8-h to 12-h shifts, separately assessing outcomes during both day and night shifts and in the home environment. None of the prospective studies assessed burnout, and the few that assessed sleep quality and duration were based on subjective reporting. Furthermore, most studies were performed over two decades ago. In 2014–2015, we had the opportunity to perform such a naturalistic pilot field study in airline ground crew managers. We tested the feasibility of a transition from a three-shift roster (day shift: 07:00–16:00, evening shift: 16:00–23:00, and night shift: 23:00–07:00 hours), hereafter, the 8-h shift roster, to a two-shift roster (day shift: 08:00–20:00 and night shift: 20:00–08:00), hereafter, the 12-h shift roster. 

The objective of the current study was to prospectively compare burnout, objective and subjective measures of sleep quality and duration, and sleepiness during day and night shifts in airline ground crew managers in the transition from 8-h to 12-h shift rosters (see Tables 4 and 5). As previous studies of managerial and low-strain occupations demonstrated a preference to a shorter and condensed workweek despite the hardship of enduring long work shifts, we hypothesized that: 

(1) Shift length is associated with burnout, self-reported sleep quality, and objective measures of sleep duration and efficiency. Compared to the 8-h shift roster, we expected to observe decreased burnout, improved sleep quality, and longer sleep duration and efficiency following the transition to a 12-h shift roster. 

(2) Shift length is associated with the level of sleepiness recorded during day and night shifts. We expected to observe that sleepiness would increase following the transition from an 8-h to a 12-h shift roster. 

## 2. Results 

### 2.1. Background Variables

Thirty-nine participants (20 males and 19 females) completed the study (mean ± standard deviation for age: 38.94 ± 8.24; body mass index (BMI): 24.72 ± 3.28; education in years: 14.72 ± 2.06; work seniority in years: 13.92 ± 7.05). No significant gender differences were found in age, education, or seniority, whereas BMI was higher (as expected) in males. 

### 2.2. Confounding Variables: Napping and Caffeine 

#### 2.2.1. Napping during the 12-h Night Shift

During the 8-h night shifts, participants did not nap. Following the transition to the 12-h roster, however, 36% reported inadvertent naps (not in compliance with the study protocol) that occurred during the period with the fewest work demands, between 03:00–04:00 at night. In order to account for this confounder, we assessed differences between nappers and non-nappers for all outcome variables.

#### 2.2.2. Caffeine Intake during 8/12-h Night Shifts

There was a statistically significant difference in caffeine intake between the two night shifts (8-h/12-h). Analysis of caffeine across the same hours revealed that on the 12-h night shift, workers drank more caffeinated beverages than on the 8-h shift (mean ± SD: 3.2 ± 1.1 cups on 12-h shift versus 0.27 ± 0.2 on 8-h shift, *t* = 26.23, *p* < 0.001). As the average amount of reported caffeine consumption was markedly higher on the 12-h compared to the 8-h shift, we conservatively controlled for caffeinated beverages in the full model (see below) by evaluating the effect of caffeine at 0.5 cups of caffeine per hour. Sensitivity analysis assessing different amounts per hour (0.25–1.5 cups) did not significantly change the findings. 

### 2.3. Burnout 

Significant differences in burnout were found for shift (Table 1), with decreased burnout during the 12-h shift roster in all three subscales and the total score: physical strength (*p* = 0.001), vitality thinking (*p* = 0.016), mental energy (*p* = 0.022), and total score (*p* = 0.002), indicating lower burnout during the 12-h as compared to the 8-h shift rosters. Assessing differences between nappers and non-nappers during the 8-h shift roster, nappers reported a significantly higher level of total burnout than non-nappers (3.61 ± 1.02 versus 2.82 ± 0.95 respectively, *p* = 0.021), while no significant differences were found during the 12-h shift roster (2.25 ± 0.98 versus 2.41 ± 0.96 respectively).

### 2.4. Sleep Quality (PSQI and Actigraphy)

**Figure 1:**
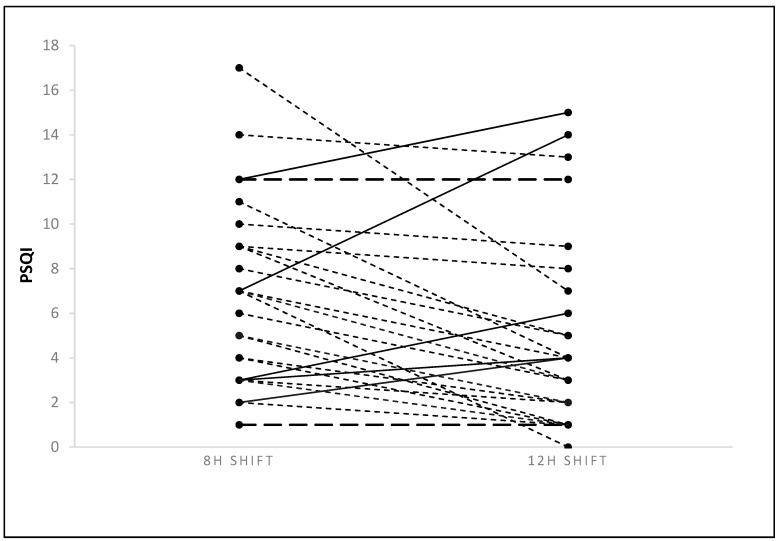
Changes in individual participants’ total PSQI scores from 8- to 12-h shift rosters. Continuous line: increased; dotted line: decreased; and dashed line: unchanged PSQI total score.

Subjective sleep quality improved based on a decrease in the total PSQI score from a mean ± SD of 6.51 ± 3.88 in the 8-h to 4.61 ± 4.07 in the 12-h shift roster (*p* = 0.003), respectively (see Figure 1 and Table 1). Based on the Pittsburgh Sleep Quality Index (PSQI) cutoff score of >5 for poor sleep quality, the rate of participants exceeding the cutoff declined from 75% to 42% in the 8-h compared to the 12-h shift rosters, respectively (*p* = 0.02). 

As measured by actigraphy, no differences were observed for any of the sleep measures during the main sleep episodes throughout the roster; however, naps beyond the main sleep periods were longer [time in bed (TIB) and total sleep time (TST)] and sleep efficiency (SE) was higher in the 12-h compared to the 8-h shift rosters (Table 2). These findings indicate improved subjective sleep quality and improved objective sleep quality at home during naps during the 12-h as compared to the 8-h shift rosters.

No differences were found between nappers versus non-nappers for the PSQI-total score during the 8-h (6.38 ± 4.27 versus 6.58 ± 3.32) and the 12-h (3.63 ± 2.82 versus 5.79 ± 4.7) shift rosters, respectively. No differences were observed between nappers and non-nappers during the 8-h shift roster in any of the objective sleep measures. During the 12-h shift roster, sleep efficiency was higher for the non-nappers (89.31 ± 5.25) than for the nappers (80.53 ± 7.96, *p* < 0.016, with Bonferroni correction). These findings demonstrate that nappers experience lower objective sleep efficiency at home during the 12-h shift rosters compared to non-nappers. 

### 2.5. Sleepiness

#### 2.5.1. Day Shift Sleepiness by Shift and Hour 

**Figure 2:**
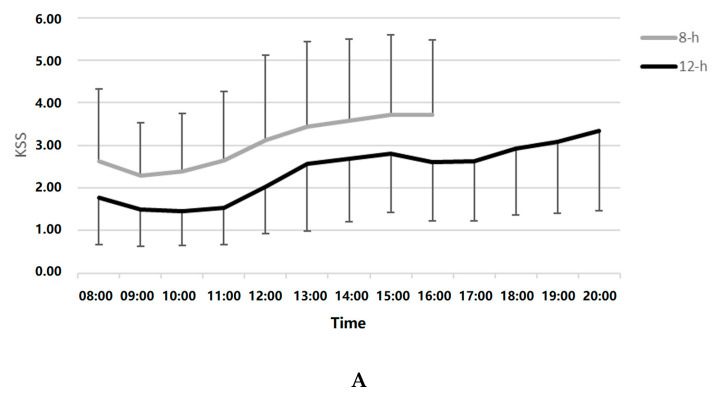

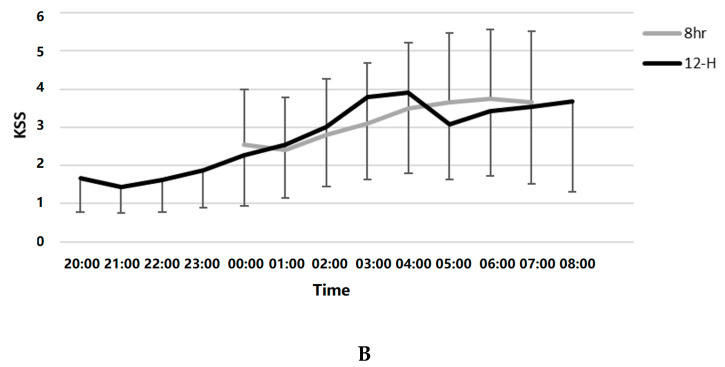
(**A**). Day shift sleepiness (Karolinska Sleepiness Scale; KSS) during the 8- versus the 12-h shift rosters. (**B**). Night shift sleepiness (KSS) during the 8- versus the 12-h shift rosters.

As the 12-h shift started at 08:00, and the 8-h shift started at 07:00, mixed model analyses for sleepiness during day shifts were performed both by clock time (starting at 08:00) and by number of hours from the beginning of the shift. As results were highly similar (near-identical), we reported clock time only. Tests of within- subject effects showed main effects for shift (F_(1,116)_ = 19.75, *p* < 0.001), and hour (F_(8,403)_ = 6.92, *p* < 0.001) with no interaction. Mean ± standard error (SE) was 3.14 ±0.19 for 8-h and 2.05 ± 0.20 for 12-h. Sleepiness levels between 08:00–11:00 were significantly lower than between 12:00–16:00 (see Figure 2A). There were no significant differences in caffeine consumption between 8-h versus 12-h day shifts (mean 0.32 ± 0.25 cups on 12-h versus 0.27 ± 0.23 on 8-h shifts) or in sleepiness between nappers and non-nappers during either the 8-h (nappers: 3.75 ± 1.91; non-nappers: 2.65 ± 0.86) or the 12-h (nappers: 2.30 ± 1.01; non-nappers: 1.92 ± 0.82) day shifts. Therefore, we did not control for caffeine or naps. Findings indicate that sleepiness increased throughout both day shifts and that sleepiness was reduced following the transition from 8-h to 12-h shifts. 

#### 2.5.2. Night Shift Sleepiness by Shift and Hour

No significant differences were found in sleepiness between the two night shifts recorded during each roster; therefore, hourly Karolinska Sleepiness Scale (KSS) was averaged over the two nights in each roster. Mixed models analysis was performed between 00:00–07:00, the clock hours that were included in both 8-h and 12-h shifts. Tests of within-subject effects showed a significant main effect for hour (F_(7,432)_ = 6.95, *p* < 0.001) but no main effect for shift (F_(1,136)_ = 0.03, *p* = 0.87), and a main effect for the hour*shift interaction (F_(7,412)_ = 2.40, *p* = 0.02; Figure 2B). Sleepiness levels between 00:00–01:00 were significantly lower than those between 03:00–07:00. Post hoc testing of the hour*shift interaction revealed no statistically significant hourly differences between shifts, with the exception of 03:00, when subjects were sleepier on the 12-h shift (mean ± SE KSS during 8-h: 3.10 ± 0.27 versus 12-h: 3.88 ± 0.31, *p* = 0.052). 

##### Night Shift Sleepiness by Shift, Hour, Nap, and Caffeine 

Due to the confounding effects of caffeine intake and inadvertent naps during the 12-h shifts only (see Section 2.2), we adjusted the night shift sleepiness model for both caffeine and naps. In the full model including all predictors (Table 3), main effects were found for hour (F_(7,355)_ = 2.13, *p* = 0.04), for shift (F_(1,226)_ = 184.51, *p* < 0.001), for nap (F_(1,78)_ = 9.81, *p* = 0.002), and for caffeine (F_(1,388)_ = 46.51, *p* < 0.001). There were significant two-way interactions for hour*shift (F_(7,344)_ = 2.24, *p* = 0.03), hour*nap (F_(7,359)_ = 2.10, *p* = 0.04), shift*caffeine (F_(1,386)_ = 76.07, *p* < 0.001), and a significant three-way shift*nap*caffeine interaction (F(1, 325) = 7.26, *p* < 0.001). No other two-way and three-way interactions were found. 

For shift, workers were sleepier on the 8-h shift than on the 12-h shift. For hour, pairwise comparisons showed that workers were significantly less sleepy at 00:00 than at 06:00 and at 01:00 than between 04:00–07:00. Nappers were sleepier than non-nappers. Increased caffeine consumption was associated with increased sleepiness (β = 0.91 ± 0.05, *p* < 0.001). The shift*hour interaction showed that workers were less sleepy on the 12-h shift than on the 8-h shift at all hours; however, sleepiness increased on the 8-h shift such that workers were less sleepy from 00:00 to 03:00 than from 04:00 to 07:00, whereas no differences were found in sleepiness throughout the night on the 12-h shift. The hour*nap interaction showed that nappers were sleepier than non-nappers between 02:00 to 07:00 but not from 00:00 to 01:00. The shift*caffeine interaction indicated that increased caffeine intake was associated with decreased sleepiness on the 8-h but not on the 12-h shift (β = −0.86 ± 0.19, *p* < 0.001). 

**Figure 3:**
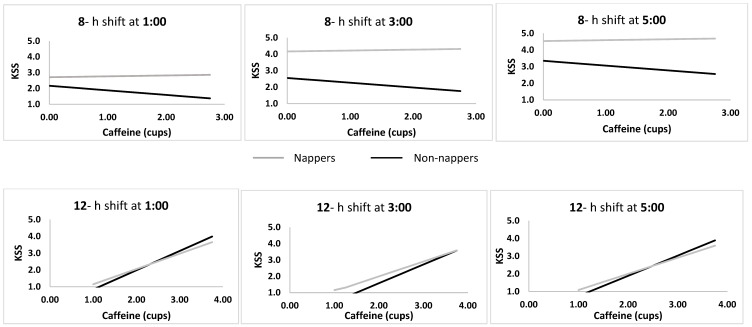
Associations between cups of caffeine and sleepiness in nappers versus non-nappers during 8-h versus 12-h night shifts at 01:00, 03:00, and 05:00.

The shift*nap*caffeine interaction showed that increased caffeine intake was related to increased sleepiness on the 12-h shift for nappers and non-nappers and to decreased sleepiness on the 8-h shift only in the non-nappers. Figure 3 shows the full model at 01:00, 03:00, and 05:00, including caffeine and nap groups in 8-h versus 12-h shift rosters. The full model suggests that behavioral strategies aimed to combat sleepiness, i.e., caffeine intake and naps, interact with shift length, thus caffeine is associated with increased (rather than decreased) sleepiness during the long (12-h) shift, whereas it is associated with decreased sleepiness during the 8-h shift for non-nappers only. 

## 3. Discussion

When comparing functional outcomes due to transitions in the length of shifts, it is important to consider additional changes that are implemented in connection with the transition. In order to maintain a comparable number of weekly work hours, 12-h shift rosters in effect compress the number of work days in the week [21]. Other changes include shift start time [8] and breaks and rest periods [6]. These and other changes may be considered necessary adaptations in the workplace, but they challenge research efforts to tease apart the effects of the separate constituents on relevant functional outcomes. Indeed, reviews comparing functional outcomes based on shift length have stressed the need to comprehensively explore the complex interactions between the various factors [7,21]. 

In the present study, burnout was lower and subjective sleep quality was higher following the transition from the 8-h to the 12- shift roster. Sleepiness increased throughout both day and night shifts and was higher during the 8-h than the 12-h day and night shifts. Sleepiness increased throughout the 8-h night shift, whereas during the 12-h night shift, sleepiness was attenuated, likely due to inadvertent napping of some of the participants. 

In line with our first hypothesis, burnout on all three subscales and total score improved, as did subjective sleep quality, following the transition from the 8-h to the 12-h shift roster. The rate of workers who exceeded the cutoff score for poor sleep quality was lower in the 12-h than in the 8-h shift roster. These results indicate decreased burnout and better sleep quality following the transition from the 8-h to the 12-h shift rosters. However, actigraphy-based sleep measures did not differ between rosters, with the exception of naps outside the main sleep periods, which showed improved sleep measures (longer time in bed, longer sleep duration, and higher sleep efficiency) during the 12-h shift roster. 

Interestingly, 36% of workers reported inadvertent naps during the 12-h night shift, between 03:00–04:00 at night. To account for this potential confounder, we compared all outcome measures in nappers and non-nappers during both rosters. Burnout was higher among the nappers during the 8-h but not the 12-h shift roster, whereas sleep efficiency (measured at home) was higher among non-nappers compared to nappers during the 12-h shift roster only. These findings suggest that nappers and non-nappers are qualitatively different in their adaptation strategies to shiftwork. Nappers may have more difficulty adjusting to the short 8-h shift roster, as indicated by increased reported burnout; however, during the 12-h shift roster, burnout was no longer higher in nappers than in non-nappers. Furthermore, during the 12-h night shift, nappers took advantage of the opportunity to nap, yet their sleep efficiency at home was compromised. It is likely that individual differences that are associated with adaptation to shiftwork, such as age, chronotype, cognitive arousal, responsibilities, and sleep opportunities at home, may underlie these different adaptation strategies [22,23]. 

Our hypothesis regarding the association between sleepiness and shift length was not supported. During the day shift, sleepiness was lower during the 12-h shift than during the 8-h shift. During the night shift, shift length interacted with hour such that during the 8-h shift, sleepiness initially decreased (at 01:00) and subsequently increased linearly, whereas during the 12-h shift, a quadratic function was observed, thus sleepiness showed an increase followed by a plateau, a decrease, and finally, no change. This decrease in sleepiness in the 12-h shift is likely due to the inadvertent naps reported by some of the workers (36%) (despite being clearly non-adherent to study protocol). However, findings also show increased sleepiness in nappers compared to non-nappers, particularly between 02:00–07:00. In a previous study comparing sleepiness and performance with and without a 30-minute nap in a simulated night shift, alertness and performance improved in the nap compared to the no-nap condition following an initial decline due to sleep inertia following the nap [24]. As napping was not part of the study design, and as only about one third of our sample reported napping, and it was only during the 12-h shift, we are unable to draw any firm conclusions regarding the temporal dynamics of sleepiness with and without naps during 8-h and 12-h shifts. 

We have previously demonstrated the efficacy of naps during the night shift on sleepiness and performance [25]. In a study of shift working hospital nurses, a short scheduled nap benefited both sleepiness and performance regardless of individual differences such as age and chronotype [25]. In the present study, workers were not expected to nap; however, it appears that nappers were compelled to do so, likely due to increased sleepiness compared to non-nappers. 

Another common strategy to cope with sleepiness during the night shift is caffeine consumption [26]. In the present study, dramatically higher caffeine consumption during the 12-h as compared to the 8-h night shift may indicate that workers anticipated and experienced greater sleepiness during the 12-h shift due to the extended hours that began three hours earlier than the 8-h shift and attempted to battle this sleepiness by increasing their caffeine consumption. However, when evaluating the relationships between caffeine and sleepiness in nappers and non-nappers, we find that increased caffeine was associated with reduced sleepiness only in the non-nappers during the 8-h shift, whereas sleepiness increased with caffeine consumption in both nappers and non-nappers during the 12-h shift, suggesting that caffeine was not effective in combating sleepiness. Whether these findings suggest individual differences in sleepiness, anticipation, behavioral adaptation to shiftwork, or other individual trait-like features is currently unclear. Individual trait-like vulnerability to sleep loss has been suggested in experimentally induced sleep deprivation protocols [27] and in nighttime shiftwork studies [22,28].

This study has some limitations. Due to management considerations of the airline company, we were not able to conduct a crossover design, and therefore order effects (i.e., a shift from 8- to 12-h versus a shift from 12- to 8-h shift rosters) could not be assessed. Furthermore, self-report measures are subject to bias, and in the present study, reported caffeine consumption was particularly high during the 12-h shift. Nevertheless, controlling for caffeine consumption did not substantially affect the main findings. We also cannot rule out the possibility that some participants failed to report inadvertent napping. Additionally, the day shift began one hour later, at 08:00 rather than 07:00 during the 12-h roster. Although we found no differences in outcome measures when computing by clock time rather than by shift start time, it is likely that any differences in outcome measures may be attributed to the additional hour at home during the day, which may be devoted to family activities or an additional hour of sleep, rather than to the difference in shift length. Another limitation is the small number of workers we followed. Thus, our results should be cautiously interpreted, as they may not be indicative of the true effect due to low statistical power. Finally, participants may have been subject to expectation bias, i.e., their improved self-report assessments following the transition to the 12-h shift roster may reflect expectations of the organization and the work environment. Anecdotal evidence obtained by incidental conversations between participants and members of the research team suggested that most of the participants were in favor of the transition. However, it is not possible to determine whether or not these reports were biased by organizational considerations. 

Notably, any transition in shift length ultimately affects several other parameters in the work schedule, e.g., start times of each of the shifts, number of shifts per 24 hours, number of work shifts per week, number of commutes per week, order of scheduled shifts and days off, etc. Therefore, it is not entirely possible to separate the distinct characteristics of the two rosters and their relative effects on study outcomes. These limitations are inherent to field studies, which nevertheless provide valuable insights to management, policy makers, and employees. 

In conclusion, the transition from 8-h to 12-h shift rosters was associated with lower burnout, improved subjective evaluation of sleep quality, and improved quality of naps in the home environment. Sleepiness was lower during the 12-h compared to the 8-h shift roster during both day and night shifts. The effects of shift on sleepiness interacted with caffeine and napping, suggesting that behavioral strategies aimed to combat night shift sleepiness differentially affected sleepiness depending on shift length. Findings highlight that in addition to factors inherent to scheduling changes, individual differences and behavioral choices should also be taken into consideration as factors that contribute to the outcomes of scheduling changes in the work environment.

## 4. Materials and Methods

### 4.1. Study Design 

The study was a within-subject prospective repeated measures design comparing measures of burnout, sleep, and sleepiness during 8-h versus 12-h shift rosters. As the study was performed in a real-life setting, it was not possible to counterbalance. All participants were initially assessed during their usual 8-h shift roster and reassessed three months after the transition to a 12-h shift roster. 

### 4.2. Participants

Thirty-nine permanent employees (19 men and 20 women) were recruited from airline ground crew managers at the Ben-Gurion International Airport. All employees worked on rotating shifts full-time and had at least one year of experience in shiftwork.

Exclusion criteria included active chronic disease affecting daily functioning, regular use of medication that affects level of alertness, employees with a child ≤1 year of age, and pregnancy. The ethics committee at Haifa University approved this study (approval # 026/14), and all participants signed informed consent.

### 4.3. Measures

**1. Shirom Melamed Burnout Measure (SMBM)** [29,30]. Burnout levels were measured using a 14-item questionnaire that included three subscales: physical fatigue, emotional exhaustion, and cognitive weariness. The participants were asked to rate their feelings during the past month on a scale of 1 (almost never) to 7 (almost always); a higher score indicated higher levels of burnout (Cronbach’s alpha 0.94 and 0.95 for 8- versus 12-h shift rosters, respectively).

**2. Pittsburgh Sleep Quality Index (PSQI)** [31,32]. The self-report questionnaire included 19 items grouped into 7 subscales on a 4-point scale (0–3, including subjective sleep quality, sleep latency, sleep duration, sleep efficiency, sleep disorders, use of sleep medications, and daily function) and summed to obtain a total score (total score >5 indicates poor sleep quality). Cronbach’s alpha was 0.71 and 0.83 for 8- versus 12-h shift rosters, respectively. In the current study, we used the total score as a continuous measure, and the cutoff score was used to dichotomize participants with/without poor sleep quality. 

**3. Activity monitoring:** Objective sleep patterns were measured using an activity monitoring device (Actiwatch 2, Phillips Respironics). This small wrist-worn device measures sleep patterns continuously in the natural environment and provides objective data for sleep patterns. Actiwatch output included 3 averaged variables for major sleep episodes and naps, as measured by the Actiwatch algorithm: time in bed (TIB, determined by event markers provided by participants when retiring to and getting out of bed), total sleep time (TST, hours from sleep onset until wake-up time), and sleep efficiency (SE, the percentage of sleep minutes out of time in bed).

**4. Karolinska Sleepiness Scale (KSS)** [33]. Participants ranked their level of sleepiness from 1 to 9: 1 = very alert; 3 = alert; 5 = neither alert nor sleepy; 7 = sleepy, but no effort required to stay awake; 9 = very sleepy, great effort required to stay awake. The KSS is widely used and has high content validity.

**5. Caffeine intake:** To control for the alerting effects of caffeine, participants reported their hourly caffeine consumption during the day and the night shift (number of cups of caffeinated beverages).

### 4.4. Procedure

The 8-shift roster included 11 days and nights, and the 12-hour shift roster included 7 days and nights (see Table 4 and Table 5). All participants were first tested during their usual 8-h shift roster and again under the 12-h shift roster after three months following the transition from 8- to 12-h shift rosters. Burnout (SMBM) and sleep quality (PSQI) measurements were completed once at the beginning of data collection for each of the two rosters (8/12). Participants wore the Actiwatch for the continuous collection of sleep measures during the full respective rosters. Subjective sleepiness (KSS) was measured hourly throughout two night shifts and one day shift per roster. 

### 4.5. Data Analysis

Statistical analyses were performed using the IBM SPSS Statistics version 21. To assess differences in sleep and burnout measures in 8-h and 12-h shift rosters, differences in the 3 burnout subscales were tested using repeated measures analysis, and differences in burnout and subjective sleep quality total scores were tested using paired t-tests. The rate of participants exceeding the PSQI cutoff score between 8-h and 12-h shift rosters were tested by chi-square. Differences in objective sleep patterns were tested using repeated measures analysis. Differences in sleepiness during day and night shifts in both rounds were tested using repeated measures analysis of the overlapping hours. Since there were no significant differences between the two recorded nights in sleepiness, a mean was calculated for each overlapping hour in both night shifts per roster. Mixed models were performed to assess differences in sleepiness levels by hour (clock time), shift (8/12), nap (nappers/non-nappers), and caffeine (number of cups of caffeinated beverages per hour), for parallel hours of both shift rosters (night shift: 00:00–07:00; day shift: 08:00–16:00).

## Figures and Tables

**Table 1:** Self-reported measures of burnout and sleep quality: comparison between 8- versus 12-hour shift rosters.

	8-h Shift	12-h Shift	Statistic, *p*-Value, Effect Size		
**Burnout**					
Physical strength	3.91 (1.36)	2.88 (1.15)	F_(1,30)_ = 13.55	*p = 0.001*	η^2^ = 0.93
Vitality thinking	2.61 (1.11)	2.06 (1.00)	F_(1,30)_ = 6.46	*p* = 0.016	η^2^ = 0.89
Mental energy	2.37 (1.13)	1.82 (0.93)	F_(1,30)_ = 5.79	*p* = 0.022	η^2^ = 0.15
Total score	3.12 (1.04)	2.34 (0.96)	*t* = 3.88	*p* = 0.002	η^2^ = 0.25
**Sleep Quality (Pittsburgh Sleep Quality Index; PSQI)**					
Total score	6.51 (3.88)	4.61 (4.07)	*t* = 3.19	*p* = 0.003	η^2^ = 0.25
PSQI > 5	75.0%	42.1%	X^2^ = 5.56	*p* = 0.02	McNemar = 0.02

**Table 2:** Objective sleep measures (time in bed (TIB), total sleep time (TST), and sleep efficiency (SE)) by shift roster (8- versus 12-h).

	8-h Shift	12-h Shift	Statistic, *p*-Value, Effect Size		
***Day sleep after night shift***					
Time in Bed (hours)	(1.34) 5.34	(1.51) 4.59	F_(1,21)_ = 3.38	*p* = 0.08	η^2^ = 0.139
Total Sleep Time (hours)	(1.16) 4.44	(1.47) 3.87	F_(1,21)_ = 2.29	*p* = 0.14	η^2 =^ 0.098
Sleep Efficiency (%)	(9.13) 82.94	(8.19) 83.72	F_(1,21)_ = 0.14	*p* = 0.71	η^2^ = 0.007
***Night sleep after day shift***					
Time in Bed (hours)	(1.26) 6.34	(1.31) 6.55	F_(1,13)_ = 0.204	*p* = 0.65	η^2^ = 0.015
Total Sleep Time (hours)	(1.25) 5.44	(1.18) 5.42	F_(1,13)_ = 0.002	*p* = 0.96	η^2^ = 0.0
Sleep Efficiency (%)	(6.20) 82.60	(6.88) 82.63	F_(1,13)_ = 0.001	*p* = 0.98	η^2^ = 0.0
***Naps (other than main sleep period) during the roster***					
Time in Bed (hours)	(0.71) 2.1	(0.74) 2.59	F_(1,9)_ = 5.7	*p* = 0.04	η^2^ = 0.388
Total Sleep Time (hours)	(0.51) 1.6	(0.73) 2.08	F_(1,9)_ = 6.47	*p* = 0.03	η^2^ = 0.418
Sleep Efficiency (%)	(7.41) 72.61	(9.47) 79.78	F_(1,9)_ = 7.57	*p* = 0.02	η^2^ = 0.457

**Table 3:** Mixed model for sleepiness during the night shift by Shift, Hour, Nap, and Caffeine consumption.

	Mean	SE	F(df)	*p*
**Shift**			184.50 (1,226)	<0.001
8-h	3.09	0.14		
12-h	0.47	0.20		
**Hour**			2.13 (7,355)	*p* = 0.04
00:00	1.52	0.15		
01:00	1.43	0.16		
02:00	1.61	0.16		
03:00	1.75	0.17		
04:00	1.94	0.17		
05:00	1.99	0.16		
06:00	2.05	0.17		
07:00	1.97	0.17		
**Nap**			9.81 (1,78)	*p* = 0.002
Nappers	2.19	0.20		
Non-nappers	1.37	0.16		
**Caffeine Intake**	0.91*	0.05	17.7 (438)	<0.001

* Beta coefficient.

**Table 4:** 8-h shift roster structure.

Day Shift	Day Shift	Day Shift	Night Shift	Night Shift	Night Shift	Free Day	Free Day	Evening Shift	Evening Shift	Evening Shift
Start of roster		Day assessment	Night 1 assessment		Night 2 assessment					End of roster

**Table 5:** 12-h shift roster structure.

Day Shift	Night Shift	Free Day	Free Day	Day Shift	Night Shift	Free Day
Start of roster	Night 1 assessment			Day assessment	Night 2 assessment	End of roster

## Abbreviations

8-h	8-hour
12-h	12-hour
SMBM	Shirom-Melamed Burnout Measure
PSQI	Pittsburgh Sleep Quality Index
KSS	Karolinska Sleepiness Scale
